# Special Issue “Experimental and Clinical Advances in Skin Grafting”

**DOI:** 10.3390/jcm12103540

**Published:** 2023-05-18

**Authors:** Joachim N. Meuli, Pietro G. di Summa

**Affiliations:** Department of Plastic and Hand Surgery, Lausanne University Hospital (CHUV), 1011 Lausanne, Switzerland; joachim.meuli@chuv.ch

## 1. Introduction

Skin grafting is one of the oldest ways to treat soft-tissue defects. In the current reconstructive surgery training, students and residents learn that it is the first surgical step of the “reconstructive ladder”, just above conservative treatment and healing by secondary intention.

However, despite this apparent simplicity, skin grafting remains a first choice in many situations and settings in which more elaborate surgical techniques are either not required or not available. 

Reports exist of skin grafting taking place more than 2500 years ago in Egyptian and Hindu societies [1], but records were patchy at best until the advent of contemporary medicine and surgery in the late 1800s, when Louis Léopold Ollier and Karl Thiersch described split-thickness grafts, Jacques Louis Reverdin described “epidermic grafting” and John Reissberg Wolfe and Fedor Krause described full-thickness grafting.

Since these reports, much has been discovered regarding the processes of imbibition, inosculation and revascularization of the skin, but the separation between split-thickness (of variable thickness) and full-thickness skin is still relevant nowadays. Split-thickness skin grafts are used to cover large defects, as their donor site heals spontaneously and the grafts can be processed to extend their coverage, whereas full-thickness skin graft are used for the reconstruction of sensitive areas and for scar corrections.

The techniques used nowadays are not very different from the descriptions made 150 years ago. Some tools and devices have been improved, some refinements introduced and some forgotten techniques (such as the MEEK technique [2]) have been reintroduced, but the overall concepts remain.

### 1.1. Is There Anything Reasonably New?

Despite few changes in the techniques themselves, several attempts have been made in the last decades to address specific but frequent issues.

The limit in the size of skin grafts has been a major issue from the beginning, especially for massively burned patients, and two approaches were developed to address it. The first focused on obtaining more time for skin grafting, i.e., on the temporary coverage of large body surfaces after debridement if split thickness is not directly available. This occurs sometimes in large burns when all available donor sites are used at once. Coverage with allogenic cadaveric skin was widely used for this and is still performed in several centers worldwide. In parallel, new products such as acellular fish skin (Nile talapia or Atlantic cod), originally brought to the market as solutions for difficult-to-treat wounds, are now being tested for this use. However, the results of the last registered trial (NCT03984331), completed in September 2021, have not shown any difference between cadaveric skin and fish skin coverage, followed by skin grafting, in regards to time to wound healing, quality of wound healing and Vancouver Scar Scale score [3].

Instead of time, the other approach focused on increasing the supply of skin to be grafted. The invention of cultured epithelial autografts by Rheinwald and Green in 1975 revolutionized the care for massively burned patients by allowing the coverage of burns reaching >90% of total body surface area. These products were themselves refined in cultured dermoepithelial autografts (CDEA) when a team added fibroblasts to keratinocytes in the early 1990s to improve skin laxity and appearance with good results. The combination of both cultured autografts with standard split thickness grafting permitted a significant improvement in skin graft take rate in massively burned patients [4]. More recently, devices that permit autologous skin cell suspension and spraying (ReCell) were developed and brought to the market, further expanding the tools available to surgeons [5].

Another issue was the difference in texture between grafted and normal skin, as well as the lack of laxity. This issue was addressed by the development of dermal substitutes such as Matriderm, Integra, AlloDerm, NovoSorb and many others. These products present some differences, but all aim to provide a dermal support for skin grafts or for epidermal cell seeding. Using these products before definite coverage with classical skin grafting (or at the same time for Matriderm) allows for the recreation of a dermoepidermal junction, which provides critical stability and shear stress resistance [6].

The issue of graft failure on difficult-to-treat wounds such as diabetic foot ulcers has been addressed by several means. Some approaches focused on optimizing the wound bed preparation, while others tried to secure skin grafts to improve resistance (Fibrin sealants such as Artiss). A classic example is the now-widespread use of negative wound pressure therapy both for wound bed preparation and to secure skin grafts in place once performed [7].

### 1.2. What Comes Next?

Innovation in skin grafting is ongoing and is still mainly focused on the abovementioned issues. 

Cultured dermoepithelial autografts have been further refined to the point where anatomical body parts such as a whole hand can be grown as a single unit, offering a significant potential to diminish scarring and improve texture [8]. Some groups are developing the next generation of cultured autografts, either by distributing dermoepithelial autografts onto three-dimensional scaffolds (denovoSkin) or by including stem cells in order to recreate a full-thickness skin substitute that includes adipose, melanocytic, nervous, lymphatic, pilosebaceous and vascular structures [9,10]. After all, normal human skin contains many more cell types than just fibroblasts and keratinocytes. Including these cells allows the capture of critical signaling functions. A critical aspect here is the induction of rapid angiogenesis, as vascularization is essential for the survival of these cells which seem to have higher metabolic requirements than keratinocytes and fibroblasts. Some groups are also attempting to automatize the process of skin production, either through large-scale production or through 3D-printing technologies. While exciting, with results such as the generation of 100 cm^2^ of skin in 35 min [11] or the demonstration that handheld bioprinters could improve the re-epithelialization of full-thickness burns, these techniques are not yet mature for application in human beings.

Dermal substitutes are further refined as well, with groups investigating oxygen species–degradable polythioketal urethane foams or gelatin-based scaffolds as alternatives to the polyester foams currently used [12]. These new structures were shown to induce less foreign body response and inflammation and to improve neo-vascularization in animal models, offering an exciting perspective for future clinical applications.

Lastly, genetically engineered skin grafts are being developed at several research institutes around the world, using viral transfection, for example. These aim to tackle all the issues at once in order to produce fully functional skin, identical to native tissues and transplantable to any individual. Combined with current genome editing tools such as the CRISPR-Cas9 system, such genetically engineered skin grafts could not only provide coverage for the wound but could also be used as a therapy for the underlying condition that caused the wound to appear in the first place, in non-traumatic cases. The major issue with such genetically engineered tissues is the same as with the direct application of growth factors to the wound: the similarity of several cellular pathways in wound healing and in oncogenesis carries a significant risk of iatrogenic-induced carcinomas. While providing thrilling opportunities for fundamental research groups, as the occurrence of cancer in an experiment can be seen as a potential target for treatment as well, this seriously limits the application and trials of such products on living humans for the time being.

This Special Issue aims to update the reader on experimental and clinical advancements in skin grafting, highlighting exciting perspectives in the plastic surgery treatments of the future.
